# Clinical characteristics and management of renal solitary fibrous tumors: a narrative review with literature-derived case evidence

**DOI:** 10.3389/fonc.2026.1894949

**Published:** 2026-08-06

**Authors:** Liang Zhao, Jiang-wei Man, Si-yu Chen, Jian-wei Yang, Jun-yang Lu, Xiao-ran Li, Pei-ting Lin, Xiao-qiang Si, Li Yang

**Affiliations:** 1 Department of Urology, The Second Hospital of Lanzhou University, Lanzhou, Gansu, China; 2 Gansu Province Clinical Research Center for Urology, Lanzhou, Gansu, China; 3 Department of Plastic Surgery, Gansu Provincial Hospital, Lanzhou, Gansu, China

**Keywords:** differential diagnosis, kidney neoplasms, prognostic risk stratification, solitary fibrous tumor, STAT6

## Abstract

**Purpose:**

Renal solitary fibrous tumor is a rare mesenchymal neoplasm with heterogeneous biological behavior. This review summarizes current evidence regarding its epidemiology, diagnosis, treatment, and clinical outcomes to support clinical management.

**Methods:**

PubMed and Web of Science were searched from database inception to September 10, 2025, for studies involving renal solitary fibrous tumors. Eligible studies included case reports, case series, and original articles providing patient-level data on clinically and histologically confirmed primary renal solitary fibrous tumors.

**Results:**

Renal solitary fibrous tumors commonly originate from the renal capsule or sinus and are frequently asymptomatic, although flank pain and hematuria may occasionally be present. Diagnostically, imaging typically reveals a well-circumscribed mass with variable enhancement patterns; however, histopathological examination remains essential for definitive diagnosis, characterized by spindle-cell proliferation and diffuse nuclear expression of STAT6. Therapeutically, complete surgical excision with negative margins constitutes the mainstay of treatment and is associated with favorable outcomes in the majority of patients. Nevertheless, approximately 10–15% of cases may develop recurrence or distant metastasis, underscoring the necessity of long-term surveillance. In the setting of advanced disease, limited data suggest that metastasectomy and selected targeted or anti-angiogenic agents may offer therapeutic benefit.

**Conclusion:**

Accurate diagnosis requires integrated clinical, radiologic, and pathologic evaluation. Complete resection remains the cornerstone of treatment, while prolonged follow-up is recommended because of the potential for delayed recurrence. Further molecular studies and collaborative investigations are needed to optimize risk stratification and therapeutic strategies.

## Introduction

Solitary fibrous tumor (SFT) is an uncommon mesenchymal neoplasm characterized by spindle-shaped cells, originally identified in the pleura and subsequently reported in multiple extrapleural locations, including the genitourinary tract (1, 2). Renal solitary fibrous tumor (rSFT) is an exceptionally rare manifestation, with the inaugural case documented by Gelb et al. in 1996 (3). Although the advent of high-resolution ultrasonography, computed tomography (CT), and magnetic resonance imaging (MRI) has significantly enhanced the detection of renal masses, rSFT remains infrequently encountered. Existing literature has addressed its epidemiology, clinical presentation, diagnostic strategies, and treatment options; nonetheless, the scarcity of reported cases and heterogeneity in study inclusion criteria continue to challenge the establishment of standardized surgical management. Notably, variables such as tumor dimensions, precise anatomical localization, and the tumor’s natural history contribute to considerable variability in therapeutic approaches among institutions (4). Renal solitary fibrous tumors (rSFTs) histologically exhibit a distinctive “patternless” architecture accompanied by hemangiopericytoma-like branching vasculature. Immunohistochemical analysis typically demonstrates diffuse CD34 positivity alongside nuclear STAT6 expression, the latter now is established as a highly sensitive and specific diagnostic marker (1, 5–13). While the majority of rSFTs demonstrate a benign clinical course, a subset possesses malignant potential, manifesting as local recurrence or distant metastasis (4, 14, 15). Currently, complete surgical excision achieving negative margins (R0 resection) remains the primary therapeutic approach and serves as the most pivotal factor influencing patient prognosis (9, 13, 16, 17). Owing to the uncommon nature of rSFTs and their potential for delayed recurrence or metastasis, prolonged postoperative monitoring is strongly advised (17, 18). This review seeks to comprehensively synthesize the clinicopathological features, diagnostic strategies, therapeutic developments, and prognostic determinants of rSFT, with the goal of informing clinical decision-making and guiding future investigative efforts.

## Methods

### Search strategy

A comprehensive search of PubMed and Web of Science was performed to retrieve studies concerning rSFTs. The search spanned from database inception to September 10, 2025. Detailed search strategies for both sources, including all keywords, MeSH terms, and Boolean operators, are presented in the **Supplementary Material**. The search was limited to English-language publications. In Web of Science, the document type was restricted to Article or Review Article. No document type filter was applied in PubMed to ensure sensitivity; retrieved records were manually screened. In addition to the systematic database search, we manually screened the reference lists of all retrieved articles and relevant reviews to identify additional eligible studies. This combined approach, which pairs systematic retrieval with citation tracking, was adopted to maximize case ascertainment for this rare tumor entity.

### Inclusion and exclusion criteria

Eligible studies met the following criteria (1): histologically confirmed primary rSFTs (2); availability of original patient-level data, including clinical presentation, pathological characteristics, treatment, or follow-up outcomes (3); publication as case reports, case series, or original research articles. Studies were excluded if they met any of the following conditions (1): reviews, editorials, conference abstracts, or commentaries without original patient data (2); solitary fibrous tumors arising from non-renal sites without clear renal primary involvement (3); insufficient clinical or pathological information for data extraction. In addition, the reference lists of all included studies were manually screened to identify further eligible articles.

### Data extraction

Two investigators independently extracted data from all eligible studies using a standardized form. Discrepancies were resolved through discussion or by consultation with a third investigator. For each case, the following information was recorded: first author and year of publication, patient age and sex, tumor location and maximal tumor dimension, immunohistochemical markers with positive expression, surgical treatment modality, and follow-up duration with documented outcomes. In instances where multiple tumors were reported in a single patient, each lesion was documented separately. All extracted data were compiled into a summarized table and a case-level detailed table (**Tables 1** and **2**) to facilitate descriptive aggregation.

**Table 1:** Summary of characteristics from 111 reported cases of renal solitary fibrous tumor.

Characteristic	Summary	Clinical significance
Total cases	111	Largest aggregated cohort to date
Age (years)	Range 3 - 85, median 52	Predominantly middle-aged; rare in children
Sex	Male: 43 (38.7%), Female: 62 (55.9%), NR: 6 (5.4%)	No significant sex predilection
Tumor size (cm)	Range 0.4 - 35.0, mean 10.1, median 8.8	Wide size variation at presentation; larger tumors (>10 cm) are associated with higher risk of adverse outcomes
Immunohistochemistry	CD34 positive: 104 (93.7%), negative: 6 (5.4%), NR: 1 (0.9%); STAT6 positive: 22 (19.8%)	CD34 is the primary diagnostic marker, positive in the vast majority of cases (93.7%). STAT6 has shown increasing utilization since 2015, reflecting its growing role as a confirmatory marker, particularly in CD34-negative or atypical cases.
Malignant cases	34 (30.6%) overall, including 16 (14.4%) with recurrence, metastasis, or disease-related death	Highlights the spectrum of biological aggressiveness; even cases without initial adverse events warrant long-term surveillance due to the potential for delayed recurrence.
Partial nephrectomy	6 cases as primary treatment; 5 with documented follow-up showed no evidence of disease.	Nephron-sparing surgery may be safe in selected patients with small, well-encapsulated, exophytic tumors in polar locations, but long-term data remain limited.
Follow-up duration (months)	Range <1 - 108, median 24	Prolonged surveillance is essential given the risk of late recurrence (up to 96 months); current median follow-up is limited, underscoring the need for longer-term studies.

NR, Not reported.

**Table 2:** Clinicopathological features of renal solitary fibrous tumors.

Case no.	Author/year	Age/sex	Tumor location	Tumor size (cm)	Immunohistochemistry(positive markers)	Treatment	Follow-up
1 (3)	Gelb et al. (1996)	48/F	Rt. Kidney (lower pole)	3.0×2.5×1.5	CD34	RN	3 days, died (respiratory event)
2 (53)	Fukunaga et al. (1997)	33/F	Rt. kidney	3×2.5×2.5	Vimentin, CD34	Rt. nephrectomy	90 months, NED
3 (53)	Fukunaga et al. (1997)	36/F	Lt. kidney	2×1.5×1.5	Vimentin, CD34	Lt. nephrectomy	12 months, NED
4 (54)	Hasegawa et al. (1999)	64/M	Kidney	4.5	Vimentin, CD34, bcl-2	Surgical excision	8 months, NED
5 (55)	Leroy et al. (2000)	66/F	Rt. kidney	9×7×6	Vimentin, CD34	Rt. RN	9 months, NED
6 (56)	Morimitsu et al. (2000)	72/F	Lt. kidney	8×7	CD34	RN	10 months, NED
7 (7)	Wang, et al. (2001)	41/M	Lt. Kidney (lower pole)	14.0×12×7	CD34, Vimentin, Collagen IV, Bcl-2	Lt. nephrectomy	48 months, NED
8 (7)	Wang, et al. (2001)	72/M	Rt. Kidney (upper pole)	13.0×9×7	CD34, Vimentin, Collagen IV, Bcl-2	Rt. nephrectomy	5 months, NED
9 (38)	Yazaki et al. (2001)	70/M	Rt. Renal pelvis	6.0×4.5×4.0	CD34, vimentin	RN	60 months, NED
10 (57)	Cortés-Gutierrez et al. (2001)	28/F	Lt. renal capsule	15×11	CD34, vimentin	Lt. RN	12 months, NED
11 (6)	Magro et al. (2002)	31/F	Rt. Kidney	8.6	Vimentin, CD34, bcl-2, CD99	Rt. RN + LND	8 months, NED
12 (58)	Llarena et al. (2003)	51/F	Bilateral kidney	Lt.: 25×15×20 Rt.: 2	CD34, Vimentina, BCL2, MIC2	Lt. RN, Rt. tumorectomy	NED
13 (59)	Yamada et al. (2004)	59/M	Lt. Kidney (upper pole)	6.8×4.4	CD34, CD99, bcl-2, vimentin	Lt. nephrectomy	48 months, NED
14 (28)	Johnson et al. (2005)	51/F	Rt. kidney	11	Vimentin, bcl-2, CD34, CD99	RN	NR
15 (60)	Yamaguchi et al., 2005	51/F	Lt. Kidney (hilar region)	10×10×5	CD34, vimentin, CD99, bcl-2	Lt. nephrectomy	NR
*16 (13)	Fine et al. (2006)	76/M	Lt. Kidney (lower pole)	12×10×7.5	CD34, Bcl-2	RN	4 months (pulmonary metastases)
17 (40)	Kohl et al. (2006)	85/F	Lt. renal hilum	4.5	CD34, Bcl-2, Vimentin	Lap. RN	NR
18 (61)	Koroku et al. (2006)	18/F	Lt. kidney (near upper calyx)	3	CD34, bcl-2, vimentin, S-100	Lap. Lt. nephrectomy	15 months, NED
19 (62)	Ferrari et al. (2006)	4/M	Rt. Kidney	8.5×6×15	NR	Rt. nephrectomy	NR
20 (63)	Bozkurt et al. (2007)	51/F	Lt. kidney	4×3.5×3.5	Vimentin, CD34, BCL-2, CD99	Lt. RN	10 months, NED
21 (64)	Constantinidis et al. (2007)	26/M	Rt. kidney	7×5.6×4	CD34, CD99, bcl-2	Rt. nephrectomy	6 months, NED
22 (65)	Znati et al. (2007)	70/M	Lt. kidney	15×12×4	CD34, CD99, Bcl-2	RN	6 months, NED
23 (66)	Hirabayashi et al. (2008)	44/F	Lt. kidney	5.8	CD34	Lt. RN	NED
24 (67)	Amano et al. (2008)	67/M	Lt. kidney	7×6.5×6	CD34, vimentin	Lap. Lt. RN	10 months, NED
*25 (68)	Magro et al. (2008)	34/F	Lt. Kidney (upper pole)	9	CD34, Bcl-2, CD99, S-100 protein	Lt. nephrectomy	21 months, NED
26 (69)	Yoneyama et al. (2009)	76/F	Rt. kidney	2.5×2.2	CD34, vimentin	Lap. Rt. RN	48 months, NED
27 (70)	Insabato et al. (2009)	70/F	Kidney	2.0	CD34	Surgical resection	31 months, NED
28 (8)	Hirano et al. (2009)	75/F	Lt. Kidney (lower pole)	4.5×3.5	CD34, Bcl-2, CD99, Vimentin	Lap. Lt. nephrectomy	9 months, NED
29 (71)	Makris et al. (2009)	35/M	Rt. Kidney (upper pole)	8	CD34, CD99, bcl2	Rt. PN and autotransplantation	NED
30 (72)	Petrella et al. (2009)	64/F	Lt. kidney	2.5	CD34, Bcl-2	Active surveillance	12 months, NED
31 (73)	Yamaguchi et al. (2010)	39/F	Lt. Kidney (lower pole)	20	CD34, bcl-2	Lt. nephrectomy	6 months, NED
32 (74)	Taxa et al. (2010)	39/F	Lt. kidney	2.5	CD34, Bcl-2, CD99	Lt. RN	12 months, NED
33 (25)	Park et al. (2011)	34/M	Rt. kidney	5.3×5×4	CD34	Rt. RN	NR
34 (39)	Naveen et al. (2011)	52/F	Rt. Kidney (upper pole)	18×9×10	Vimentin, CD34, Factor VIII, CD-99, Bcl-2	Rt. RN + thrombectomy	6 months, NED
*35 (75)	Hsieh et al., 2011	50/F	Rt. kidney	9×9×6	CD99, Vimentin	Rt. RN	30 months, NED
*36 (76)	Marzi et al. (2011)	72/F	Lt. kidney	19	Vimentin, CD99, PDGFRB, CD34	Lt. RN	15 months, NED
37 (77)	Gutiérrez-Díaz et al. (2011)	46/F	Lt. kidney	11	Vimentin, CD34, Bcl-2	Surgical resection	18 months, NED
*38 (17)	Zhao et al., 2012	56/M	Lt. Kidney (upper and lower poles)	10×8×7 (upper pole), 10×7×7 (lower pole)	CD34, CD99, Vimentin	Lt. RN	10 months, NED
*39 (78)	Guo et al., 2012	60/M	Rt. kidney	NR	CD34, CD68, vimentin	Lap. RN + metastatic tumor resection	5 months, died of metastatic disease
40 (79)	Rodríguez-Moreno et al. (2012)	47/F	Rt. renal hilum	2.6×1.7×2.8	CD34, CD99, Vimentin, Bcl2	Rt. RN	NR
*41 (80)	De Martino et al. (2012)	68/F	Lt. kidney	7	CD34, Vimentin	RN	5 months (died)
42 (81)	Fritchie et al. (2012)	55/F	Kidney	2.1	CD34	NR	39 months, NED
*43 (20)	Sfoungaristos et al. (2012)	72/M	Lt. Kidney (upper pole)	7×6	CD34, Vimentin, bcl-2	RN	36 months, recurrence;
*44 (47)	Cuello et al. (2013)	49/F	Lt. Kidney	9.8	CD34, CD99, BCL-2, Vimentin	Lt. nephrectomy, Liver Metastasectomy, Subcutaneous Interferon α2b	23 months, (At diagnosis: pleural, pulmonary, hepatic metastases)
*45 (82)	Patel et al. (2013)	43/M	Primary: Lt. kidney; Metastases: Head of pancreas, Liver	Pancreas: 5.7×5.2 Liver: 3×2.7	CD99, CD34, Bcl-2	Lt. nephrectomy (2002), Whipple + partial hepatectomy (2011)	18 months, NED
46 (4)	Khater et al. (2013)	49/F	Rt. kidney	5×5×2.5	CD34, bcl-2, vimentin	Rt. Lap.RN	NED
47 (83)	Kamath et al. (2013)	70/M	Rt. kidney	13×10×8	CD34, bcl-2, vimentin	Rt. RN	7 days, died (cardiac arrest)
48 (84)	Demirtas et al. (2013)	57/M	Lt. kidney	14×12×11	CD34, CD99	Lt. RN	26 months, NED
*49 (85)	Sasaki et al. (2013)	48/M	Rt. Kidney (upper pole)	28×18×10	CD34, vimentin, CD99	Rt. RN, Pulmonary segmentectomy + partial hepatectomy	96 months to metastasis; 12 months NED after surgery
50 (86)	Wu et al. (2013)	3/M	Lt. kidney	15×10×7	CD34, vimentin	Lt. RN and adrenalectomy	3 months, NED
*51 (87)	Mearini et al. (2014)	19/F	Lt. kidney	14.5×9×8.5	CD34, BCL2	RN + regional LND	30 months, (LN metastasis at diagnosis)
*52 (88)	Rodríguez et al. (2014)	84/F	Lt. Kidney	30.7×22.6×18.5	CD34, CD99, Bcl2, vimentin, smooth muscle actin	Lt. RN + resection of peritoneal implants	3 months, died
53 (89)	Ma et al. (2014)	35/F	Lt. kidney	0.4	CD34, Bcl-2, CD99, Vimentin	Lt. nephrectomy	15 months, NED
54 (90)	Tritschler et al (2014),	55/NR	Rt. Kidney (renal sinus)	5×7	CD34, Bcl2, CD99, vimentin	Rt. RN	NR
55 (91)	Dong et al. (2014)	71/F	Lt. kidney	4×3.5×4	CD34, CD99, bcl-2, CD34	Lap. RN	24 months, NED
*56 (92)	Wang et al. (2014)	66/F	Rt. kidney	23×18×12	CD34, CD99, Bcl-2, vimentin	RN	9 months, NED
57 (33)	Ichiyanagi et al. (2015)	41/F	Lt. Kidney (lower pole)	3.8×3.75×3.36	CD34, CD99, Bcl2, PAX8, NAB2, STAT6, GRIA2	Lap. RN	25 months, NED
58 (93)	Abeygunasekera et al. (2015)	68/F	Lt. Kidney (upper pole)	4.5	CD34, CD99, Bcl-2	Lt. Open RN	6 months, NED
59 (94)	Nakajima et al. (2015)	63/F	Rt. kidney	10×9.7	CD34	Total nephrectomy	6 months, NED
*60 (95)	Cheung et al. (2015)	49/F	Lt. Kidney (initial), Rt. Kidney (recurrent)	Initial: 16×11×9 Recurrent: 4.3	Initial: CD34; Recurrent: CD99, BCL-2, vimentin	Lt. RN + retroperitoneal LND, Robotic-assisted Lap. Rt. PN	96 months, contralateral recurrence (Rt. kidney)
61 (96)	Chen et al. (2015)	30/F	Rt. kidney	6×4.5	CD34, Bcl-2	Surgical excision	19 months, NED
62 (46)	Martini et al. (2016)	37/M	Lt. kidney	2.5×2.5	CD34	Robotic clampless PN	15 months, NED
63 (18)	Fursevich et al. (2016)	66/F	Lt. Kidney (upper pole)	9.3×7.9×9.4	CD34, BCL2, CD99	Lt. nephrectomy	24 months, NED
*64 (97)	Usuba et al. (2016)	50/M	Lt. kidney	17×11×8	CD34, Bcl-2, CD99, STAT6	RN	36 months (Local recurrence)
65 (98)	Ikeda M et al. (2017)	59/F	Rt. Kidney (lower pole)	8	CD34, CD99, bcl-2	PN	36 months, NED
66 (29)	Lalwani AK et al. (2018)	35/M	Rt. kidney	19.8×16.3×13.7	CD34, Vimentin, p53	Rt. nephrectomy	NR
67 (16)	Bacalbasa et al. (2018)	49/NR	Rt. kidney	16×14×8	CD34, BCL2, CD99, SMA, desmin, S100	Rt. nephrectomy	NR
68 (45)	Chen et al. (2018)	27/F	Rt. kidney	4×3×2.5	CD34, EMA, Bcl-2, CD99	Lap. PN	24 months, NED
69 (99)	Xie et al. (2018)	NR	Renal pelvis	4.5×3.5×2.8	CD34, Bcl-2	Transurethral resection	24–36 months, NED
70 (99)	Xie et al. (2018)	NR	Renal Pelvis	4×4×3.8	CD34, Bcl-2	Transurethral resection	24–36 months, NED
71 (99)	Xie et al. (2018)	NR	Renal pelvis	4×3.9×3.7	CD34, Bcl-2	Transurethral resection	24–36 months, NED
72 (99)	Xie et al. (2018)	NR	Renal Parenchyma and Renal Capsule	11.4×10.2×11.9 10.1×7.6×12.1	CD34, Bcl-2	Posterior Lap. renal tumorectomy	24–36 months, died
*73 (100)	Zhang et al. (2018)	62/F	Lt. Kidney (upper pole)	6.5×6×5.5	CD34, VIM, S100, EMA	Lap. RN, Partial hepatectomy	NR
74 (1)	Zaghibib S et al. (2019)	55/M	Lt. Kidney (lower pole)	4×4×3	CD34, Bcl-2	Open PN	9 months, NED
*75 (101)	Sammoud et al., 2019	53/M	Lt. kidney	13×10×8	CD34	Initial: Open Lt. RN with retroperitoneal LND. First recurrence: Open surgical excision. Second recurrence: Open resection + preop RT/CT	9 months, local recurrence + pulmonary metastasis (partial response)
*76 (37)	Argani et al. (2019)	30/F	Kidney	10	BCOR, STAT6, CD99, CD34	NR	NR
*77 (37)	Argani et al. (2019)	40/M	Kidney	18.5	BCOR, CD99	NR	NR
*78 (37)	Argani et al. (2019)	62/F	Kidney	26	BCOR, STAT6, CD34	NR	NR
*79 (37)	Argani et al. (2019)	48/M	Kidney	10.0	BCOR, STAT6, CD99	NR	NR
*80 (102)	Couture et al. (2019)	72/M	Rt. Kidney (upper pole)	6×4.5×4.0	CD99, CD34, Vimentin	Open Rt. RN + Rt. adrenalectomy	9 months (bilateral lung nodules)
81 (103)	Fu et al. (2019)	50/M	Lt. kidney	10×9×6	CD34, bcl-2	Open RN	36 months, NED
82 (103)	Fu et al. (2019)	48/F	Lt. kidney	7×6×6	CD34, bcl-2	Open RN	85 months, NED
83 (103)	Fu et al. (2019)	60/F	Rt. kidney	8×4×3	CD34, bcl-2	Lap. RN	78 months, NED
84 (103)	Fu et al. (2019)	47/M	Rt. kidney	9×4×5	CD34, bcl-2	Open RN	56 months, NED
*85 (103)	Fu et al. (2019)	44/F	Rt. kidney	14×10×8	CD34, bcl-2	Open RN	49 months, NED
86 (103)	Fu et al. (2019)	45/M	Rt. kidney	9×6×5	CD34, bcl-2	Lap. RN	44 months, NED
*87 (103)	Fu et al. (2019)	61/M	Rt. kidney	19×12×10	CD34, bcl-2	Open RN	35 months, NED
88 (103)	Fu et al. (2019)	53/M	Rt. kidney	13×12×9	CD34, bcl-2	Open RN	48 months, NED
89 (103)	Fu et al. (2019)	63/F	Lt. kidney	8×6×5	CD34, bcl-2	Lap. RN	26 months, NED
90 (103)	Fu et al. (2019)	38/M	Lt. kidney	5.3×4×3	CD34, bcl-2	Lap. RN	16 months, NED
91 (104)	Zhang et al. (2019)	45/F	Lt. Kidney (renal pelvis)	4×2.5×2	CD34, CD99, STAT6	Surgical resection	NR
92 (22)	Ramos et al. (2020)	83/M	Lt. kidney	19×17×18.7	STAT6, CD34, IGF-II	Open Lt. RN	12 months, NED
93 (105)	De Luca et al. (2020)	52/F	Rt. Kidney (lower pole)	7.4×6.3×5.8	CD34, STAT6	Lap. RN	NR
94 (106)	Kopel et al. (2020)	45/F	Rt. kidney	10.1×7.7×9.09	CD34, Bcl-2	Open Rt. RN + LND	3 months, NED
*95 (107)	Klubben et al. (2021)	67/F	Rt. kidney	25×17.3×13.3	STAT6, CD34, BCL-2	RN	Serial assessments every 3 months
*96 (30)	Almohammad et al. (2021)	35/F	Rt. Kidney, IVC, and Right Atrium thrombus	22×14×9	Supportive of malignant SFT	Rt. nephrectomy	6 months, (Progression: 4 cm right pulmonary node)
*97 (108)	Luis-Cardo et al. (2021)	66/M	Rt. kidney (renal sinus and lower pole)	35×21×14	CD34, STAT6	Open RN with preservation of ipsilateral adrenal gland	NR
*98 (109)	Izumi et al. (2022)	75/M	Lt. kidney	5.5×3.5×2.5	CD34, STAT6, bcl-2	Lap. Lt. RN	7 months, NED
99 (110)	Ganeva et al. (2022)	15/F	Lt. kidney	1.1×1.1	CD34	Lap. resection	6 months, NED
*100 (21)	Zhang et al. (2022)	68/M	Rt. Renal Pelvis	9.9×7.3	Bcl-2, CD34, VIM	Rt. nephrectomy	60 months;NED
101 (31)	Lobo A et al. (2022)	44/M	Rt. Kidney (lower pole)	19×17×12	CD34, STAT6, BCL2, CD99, Vimentin	RN	15 months, NED
*102 (11)	Zhao et al. (2022)	54/M	Rt. Kidney	5	STAT6, CD34	RN	16 months, (Progression: Pulmonary and Brain)
103 (35)	Ji et al. (2022)	34/F	Lt. kidney	4.1×4.5	STAT6, CD34, CD99, Bcl-2, Vimentin	Lap. RN	12 months, NED
104 (111)	Liu M et al. (2022)	76/M	Rt. renal pelvis	2.7×2.5×1.6	CD34, STAT6, vimentin, Bcl-2, CD99, SMA	Lap. Rt. nephroureterectomy	36 months, NED
105 (9)	Tahir et al. (2023)	39/F	Lt. Kidney (lower pole)	4.6×4.3×3	Vimentin, Bcl-2, CD99, STAT6	RN	2 months, NED
*106 (32)	Lobo et al. (2023)	48/M	Rt. Kidney (upper pole)	6.8×6.5×4	STAT6, BCOR, CD99	RN	13 months, NED
107 (42)	Saad et al. (2024)	64/F	Lt. Kidney (upper pole)	2.5	CD34, BCL2, STAT6 (spindle cells); Keratin 7, PAX8, ESR1, PGR	PN	NR
*108 (51)	Zhou et al. (2024)	51/M	Lt. kidney	19×14×11.	STAT6, CD34, IGF2	RN	1 month, NED
*109 (5)	Ramineni et al. (2024)	57/F	Lt. Kidney (lower pole)	11.5×10×8	STAT6, Vimentin, Bcl-2	Robotic-assisted RN	Close surveillance with serial CT imaging
110 (12)	Angelico et al. (2024)	31/F	Lt. Kidney (upper pole)	8.5	STAT6, CD34, Vimentin	RN	108 months
111 (12)	Angelico et al. (2024)	34/F	Rt. Kidney	9	STAT6, CD34, Vimentin, S100	RN	108 months

Rt., Right; Lt., Left; Lap., Laparoscopic; LN, Lymph node; LND, Lymph node dissection; NED, No evidence of disease; RN, Radical nephrectomy, PN, Partial nephrectomy; Preop, Preoperative; RT, Radiotherapy; CT, Chemotherapy; IVC, Inferior vena cava; NR, Not reported.

(*) indicates malignant behavior (recurrence, metastasis, or disease-related death) or pathologically confirmed malignancy.

### Epidemiology

Epidemiological evidence indicates that solitary fibrous tumors (SFTs) are spindle-cell neoplasms predominantly originating from the pleura, though they have been documented in a diverse array of extrapleural locations, including the orbit, nasal cavity, paranasal sinuses, thyroid, liver, pancreas, kidney, bladder, and prostate (14). The rSFTs are particularly uncommon, generally presenting in individuals aged 28 to 83 years, with an average onset around 52 years; over half of reported cases occur in patients older than 40 (19). No clear sex predominance has been identified (14), and the renal capsule is most frequently regarded as the primary site of tumor origin within the kidney (20).

### Clinical presentation

The rSFTs present with highly variable clinical features. The most frequently observed manifestations include hematuria, flank or abdominal discomfort, and the detection of a palpable mass, which can closely mimic the presentation of renal cell carcinoma (4). In a subset of patients, tumor-induced overproduction of insulin-like growth factor 2 (IGF2) may result in persistent hypoglycemia, leading to syncope or altered levels of consciousness, a rare paraneoplastic phenomenon referred to as Doege Potter syndrome (21–24). Moreover, there have been isolated reports of renal SFTs being associated with complications such as acute pyelonephritis and urosepsis (4).

### Imaging findings

The rSFTs on ultrasonography generally appear as well circumscribed masses with hypoechoic or heterogeneous internal echoes (25). Unenhanced computed tomography typically demonstrates lesions with slightly higher attenuation than adjacent renal parenchyma, often enclosed by a pseudocapsule. Their heterogeneous cellular architecture and collagen content produce internal alternating density patterns. After contrast administration, these tumors present as lobulated soft tissue masses with heterogeneous enhancement, most often showing progressive or persistent contrast uptake. A central fibrous scar is a characteristic feature, appearing as a nonenhancing hypodense core on enhanced images, more frequently in benign lesions and potentially indicating a less aggressive course (26).

Magnetic resonance imaging typically shows rSFTs with low to intermediate signal intensity on both T1 and T2 sequences. T2 weighted images may display a radiating pattern with alternating very low and intermediate to high signal regions, a feature suggestive of SFT. This heterogeneity reflects underlying histology, where cellular areas intermingle with dense collagen, while higher T2 signal may indicate myxoid change or cystic degeneration. Following contrast administration, marked enhancement is common, occasionally forming a spoke wheel pattern. Necrosis, hemorrhage, and calcification are usually absent, and delayed imaging may reveal persistent enhancement (25, 27, 28).

### Histopathological findings

The rSFTs show considerable size variation, measuring 2 to 25 cm with a mean of 8.75 cm. They are typically well circumscribed, often encapsulated, and display a lobulated, firm to hard structure. Cut surfaces are usually homogeneous, gray to gray white, and commonly exhibit a whorled pattern. Necrosis, cystic change, and hemorrhage are infrequent and mainly associated with malignant forms (4, 19, 29). Some tumors contain intermixed yellow adipose and gray white fibrous areas, producing a fatty appearance (16). In certain cases, intravascular tumor thrombi may form and extend into the inferior vena cava or even the right atrium (30). Rarely, a predominantly multilocular cystic pattern is observed, characterized by thin walled cysts with minimal hemorrhagic fluid and an accompanying solid nodule, thereby expanding the known gross morphological spectrum (31).

**Figure 1:**
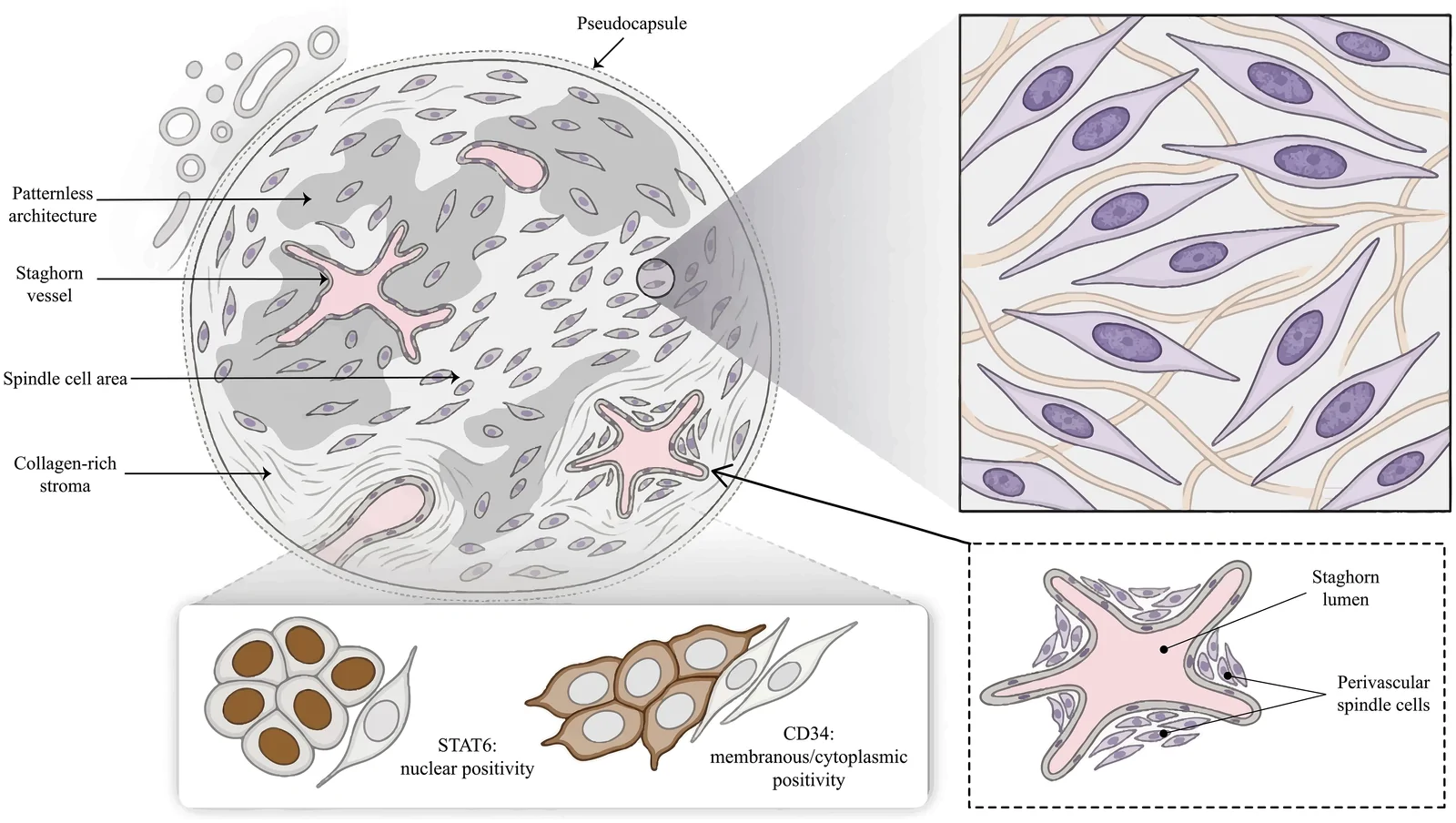
Schematic illustration of the histopathological and immunohistochemical features of renal solitary fibrous tumor.

Histologically, rSFTs are usually well circumscribed and composed of uniform spindle cells with limited cytoplasm and oval nuclei, showing minimal atypia and rare mitoses, typically fewer than four per ten high power fields. A patternless architecture predominates, with irregular admixture of spindle cells and collagen, although fascicular or storiform patterns may occur. Characteristic branching vessels resembling hemangiopericytoma, often dilated into a staghorn configuration, are commonly observed (**Figure 1**) (4–8, 25). In 1989, England et al. proposed diagnostic criteria for malignant SFTs that remain in use, including high cellularity, nuclear crowding and overlap, marked atypia with pleomorphism, hyperchromasia and conspicuous nucleoli, and increased mitotic activity defined as more than four per ten high power fields as the key quantitative indicator. Tumor necrosis, hemorrhage, and loss of CD34 and Bcl-2 expression are also included. Additional features comprise large size greater than 10 cm and sessile growth lacking a pedicle or showing a broad base (9, 10).

### Immunohistochemical findings

Immunohistochemistry is pivotal for diagnosing rSFTs. These tumors typically express CD34, STAT6, CD99, and Bcl-2, with CD34 and STAT6 as key markers, while CD99 and Bcl-2 coexpression occurs in about 70 percent of cases (1, 4, 11–13, 19). STAT6 shows high sensitivity and specificity due to NAB2-STAT6 fusion, which induces nuclear localization and strong diffuse staining. Compared with CD34, nuclear STAT6 improves diagnostic precision, especially in atypical or CD34-negative cases, including the round cell variant. Rare dedifferentiated liposarcomas may also exhibit nuclear STAT6 positivity from 12q13–15 amplification, but can be distinguished by concurrent MDM2 amplification detected by fluorescence *in situ* hybridization or immunohistochemistry (32–35).

Other immunohistochemical markers of potential diagnostic significance include vimentin, epithelial membrane antigen, and smooth muscle actin (14, 36). In addition to these traditional markers, recent investigations have highlighted BCOR expression in a subset of solitary fibrous tumors, particularly those with malignant features (37). Tumor cells typically do not exhibit immunoreactivity for S100, cytokeratins, or desmin, and when staining is observed, it is usually focal or weak (4, 9, 19, 36).

### Ultrastructural findings

Electron microscopy demonstrates that rSFTs consist predominantly of spindle-shaped cells resembling fibroblasts, with nuclei frequently presenting irregular contours and occasional peripheral chromatin condensation (3, 8, 38). The cytoplasm is rich in organelles, notably extensive rough endoplasmic reticulum and prominent Golgi apparatus, along with variable numbers of mitochondria and dispersed intermediate filaments, aligning with immunohistochemical vimentin expression. Intercellular connections are limited, manifesting only as occasional primitive junctions, with desmosome-like structures absent. Tumor cell extensions often infiltrate the surrounding collagenous stroma, which is densely embedded with abundant collagen fibers (8, 38, 39). Taken together, these ultrastructural features support the designation of solitary fibrous tumors as neoplasms exhibiting mesenchymal fibroblastic differentiation and provide a basis for differentiating them from other spindle cell tumors, including leiomyomas and schwannomas (7, 8, 39).

### Differential diagnosis

The rSFTs demonstrate marked morphological variability, requiring careful distinction from a range of both benign and malignant spindle cell neoplasms within the kidney. Among the benign lesions are fibromas, benign fibrous histiocytomas, hemangiopericytomas, leiomyomas, angiomyolipomas containing smooth muscle elements, and peripheral nerve sheath tumors, including neurofibromas and schwannomas (6, 40). All these neoplasms are composed of spindle cells, and overlapping histological characteristics with SFTs often hinder definitive diagnosis using light microscopy alone. Consequently, immunohistochemical analysis plays a critical role, as rSFTs consistently demonstrate positivity for CD34, Bcl-2, and CD99, whereas markers such as S100, SMA, Desmin, and CD117 are typically absent (6, 40, 41). In contrast, neurofibromas characteristically show strong S100 expression but are typically negative for CD34 and CD99 (41). Tumors with smooth muscle differentiation, including leiomyomas, consistently exhibit positivity for SMA and Desmin. Angiomyolipomas containing smooth muscle elements express HMB45 in addition to SMA and Desmin, yet they generally lack CD34 expression (40). Furthermore, schwannomas may display limited or focal CD34 positivity, but the presence of EMA serves as a useful marker to differentiate them from SFTs (6, 41).

Differentiating rSFTs from malignant renal neoplasms is especially crucial. Monophasic fibrous-type synovial sarcomas are defined by the t(X;18) chromosomal translocation and lack expression of both CD34 and STAT6. Sarcomatoid renal cell carcinomas consistently exhibit cytokeratin positivity. Leiomyosarcomas are distinguished by marked mitotic activity and areas of necrosis, alongside immunoreactivity for SMA and Desmin. Malignant peripheral nerve sheath tumors may show limited S100 positivity but are generally negative for CD34. Fibrosarcomas usually do not express CD34 or Bcl-2 (13). Notably, high-grade or dedifferentiated renal SFTs can occasionally demonstrate BCOR positivity (5, 37), a characteristic that overlaps with pediatric renal clear cell sarcoma (CCSK). Unlike SFTs, CCSK lacks STAT6 expression and does not harbor the NAB2-STAT6 fusion gene. Similarly, synovial sarcomas may express BCOR but are distinguished by the presence of the SS18-SSX fusion gene and absence of STAT6. Consequently, in adult patients, undifferentiated renal tumors that are BCOR-positive and either negative or only focally positive for CD34 should undergo STAT6 immunohistochemical evaluation and molecular testing to reliably exclude SFT (37). Furthermore, a subset of SFTs can present in association with Müllerian-type cysts, referred to as SFT-MC, which requires careful distinction from mixed epithelial–stromal tumors (MEST) and angiomyolipomas containing epithelial cysts (AMLEC). In SFT-MC, the stromal component consistently expresses STAT6 and CD34, while the epithelial component shows positivity for estrogen and progesterone receptors. By contrast, although the stromal elements of MEST and AMLEC may exhibit ER and PR expression, they do not express STAT6 or CD34, and their epithelial components are typically negative for hormone receptors. Synovial sarcomas, in comparison, lack expression of both STAT6 and hormone receptors (42).

Imaging studies play an essential role in the differential diagnosis of renal tumors. The rSFTs most commonly originate from the renal sinus, while renal cell carcinoma (RCC) arising in this location is exceptionally uncommon, representing only 1.2% of RCC cases in a single-center cohort. On non-contrast computed tomography, rSFTs typically present as mildly hyperdense masses surrounded by a pseudocapsule, often with partially ill-defined borders. Their enhancement pattern is generally progressive or persistent, contrasting with the characteristic rapid enhancement and washout pattern seen in RCC. Moreover, rSFTs usually displace hilar vessels without direct invasion, whereas RCC tends to exhibit vascular invasion or replacement. Other interstitial tumors of the renal sinus can likewise be differentiated based on their distinctive imaging characteristics. For example, lipomas and angiomyolipomas usually demonstrate prominent fat signals on imaging, whereas Castleman disease is characterized by arborizing calcifications. Paragangliomas often exhibit intense enhancement with delayed contrast washout and may present with areas of cystic change, necrosis, or calcification. When a renal sinus originating mass exceeds 10 cm in diameter and displays lobulated contours, heterogeneous enhancement, and patchy progressive enhancement, these features should prompt a high index of suspicion for malignant rSFT (26).

In conclusion, establishing a definitive diagnosis of rSFTs requires a comprehensive evaluation that integrates histopathological morphology, immunohistochemical markers, and radiological findings. This multidisciplinary approach is crucial for differentiating rSFTs from other benign or malignant neoplasms with overlapping morphological or immunophenotypic features, ultimately guiding appropriate clinical management and treatment planning.

### Therapies

Surgical excision remains the principal and most effective therapeutic option for rSFT, with complete removal of the lesion serving as a pivotal prognostic factor (9, 13, 16, 17). Radical nephrectomy (RN) continues to be the conventional treatment of choice, especially in cases where the tumor infiltrates the renal parenchyma or extends to adjacent anatomical structures. Attaining an en bloc resection with histologically negative margins (R0 resection) is imperative, as the presence of positive margins markedly heightens the likelihood of local recurrence and distant dissemination (13, 16). For benign SFT, radical nephrectomy generally yields excellent long-term outcomes, and most patients remain free from disease recurrence during postoperative surveillance (13).

In recent years, the development of minimally invasive surgical technologies has facilitated the adoption of nephron-sparing surgery (NSS) as a viable alternative in carefully selected patients, particularly those presenting with small, well-encapsulated, and anatomically favorable tumors. Recent reviews on robot-assisted kidney surgery have highlighted technical refinements that facilitate NSS, with patient selection considerations such as tumor size, anatomical location, and malignant potential remaining central to clinical decision-making. Regarding tumor location, the retroperitoneal approach via lower anterior access provides direct exposure of posterior tumors and is associated with reduced postoperative pain and shorter hospital stays, whereas the transperitoneal approach remains appropriate for anterior or lateral lesions. Collectively, these technical refinements have broadened the applicability of NSS and improved perioperative outcomes in renal surgery (43, 44). Both laparoscopic and robot-assisted partial nephrectomy techniques have demonstrated the ability to achieve negative surgical margins while maintaining optimal renal function. Evidence from case reports suggests that these approaches can produce satisfactory short-term oncological outcomes, with patients showing no signs of local recurrence or distant metastasis during follow-up periods extending up to two years (45, 46). However, due to the rarity of renal SFT and its estimated malignant potential of approximately 10–15%, long-term comparative evidence remains scarce. Therefore, radical resection continues to be advocated for tumors that are large in size or exhibit invasive growth patterns (18). Intraoperative frozen section examination serves as a valuable tool for assessing margin status and determining the necessary extent of resection. When malignant characteristics or positive margins are detected, conversion from partial to radical nephrectomy should be undertaken to achieve complete tumor removal (9). Given the nonspecific nature of imaging findings and the potential for sampling error in biopsies, routine preoperative percutaneous biopsy is currently not recommended as a definitive diagnostic approach (18).

Irrespective of the surgical approach adopted, diligent postoperative monitoring remains essential. While local recurrence occurs infrequently, the documented emergence of asynchronous lesions in the opposite kidney and distant metastatic sites underscores the possibility of multicentric disease development (13, 17, 18). Although standardized, evidence-based recommendations for follow-up intervals and surveillance protocols are not yet established, sustained and systematic imaging assessments over the long term are strongly recommended to facilitate early detection of recurrence and metastatic spread.

For patients with unresectable, recurrent, or metastatic renal SFT, no established systemic therapy currently exists. Available evidence is largely confined to isolated case reports and small retrospective series, and should therefore be considered hypothesis-generating rather than practice-defining. It should be noted that the efficacy data for systemic agents described below are predominantly derived from studies that included solitary fibrous tumors of various anatomical sites, their specific applicability to renal SFT requires further long-term validation. To enhance clarity, the reported systemic treatments can be grouped according to their primary mechanism of action. **①** Immunomodulatory and combination regimens. Some reports have shown that subcutaneous administration of interferon α2b can maintain disease stability for as long as 23 months (47). Furthermore, evidence suggests that the combination of temozolomide and bevacizumab demonstrates substantial antitumor efficacy in advanced SFT, achieving an objective response rate of 79% and a median progression-free survival of about 9.7 months, thereby indicating a promising therapeutic avenue for affected patients (48). **②** Cytotoxic chemotherapy. Traditional cytotoxic chemotherapy, including doxorubicin-based and ifosfamide-containing regimens, is generally administered according to treatment protocols used for soft tissue sarcomas; however, its overall therapeutic benefit remains modest. Two retrospective studies evaluating conventional chemotherapy in advanced SFT reported objective response rates ranging from 0% to 20% and median progression-free survival of approximately 4 months, indicating that disease stabilization rather than tumor regression is the predominant outcome (49, 50). **③** Targeted therapy. Current evidence indicates that tyrosine kinase inhibitors targeting platelet-derived growth factor receptor β (PDGFR-β), such as imatinib, may exert therapeutic effects in tumors harboring wild-type PDGFR-β (17). In addition to systemic therapies, radiotherapy may serve as a complementary modality for local control in patients with unresectable or recurrent lesions (17). Given the low level of available evidence, treatment decisions for advanced renal SFT should be individualized, ideally within the setting of a multidisciplinary tumor board.

In summary, complete surgical resection with histologically negative margins continues to represent the cornerstone and most effective treatment for renal SFT. Considering its intermediate malignant potential, sustained and meticulous postoperative follow-up is essential to detect recurrence or metastasis at an early stage. The therapeutic roles of nephron-sparing surgery, systemic treatments, and targeted molecular agents remain subjects of ongoing exploration. Ultimately, the development of standardized management protocols will depend on evidence derived from multicenter collaborations and rigorously designed prospective clinical trials.

### Prognosis

The rSFTs are generally benign neoplasms; however, a subset, approximately 10–15% of cases, demonstrates malignant behavior or metastatic potential (4, 14). Notably, the proportion of pathologically defined malignant tumors exceeded the rate of documented adverse clinical events in this cohort. This discrepancy indicates that histological malignancy does not invariably translate into aggressive behavior, whereas some morphologically benign tumors may still recur after prolonged intervals. Thus, pathological malignancy should be interpreted as a risk indicator rather than a definitive surrogate for clinical outcome, reinforcing the necessity of long-term surveillance irrespective of initial grading. In the fifth edition of the World Health Organization (WHO) Classification of Tumors, predictive models have been developed to assess the metastatic risk of solitary fibrous tumors (SFT), including both three-variable and modified four-variable systems. These models evaluate clinical and pathological parameters such as patient age, mitotic index, tumor size, and the presence of necrosis. Within the scoring framework, patients younger than 55 years receive 0 points, whereas those aged 55 years or older receive 1 point. Mitotic activity per 10 high-power fields is rated as 0 points for 0, 1 point for 1–3, and 2 points for 4 or more. Tumor size contributes 0 points if less than 5 cm, 1 point if less than 10 cm, 2 points if less than 15 cm, and 3 points if 15 cm or greater. Summation of these scores allows SFTs to be categorized into low-risk (0–2 points), intermediate-risk (3–4 points), or high-risk (5–6 points) groups for metastasis prediction (51). Although the WHO risk stratification model provides a standardized framework for predicting metastatic risk in solitary fibrous tumors, its direct application to renal SFT should be interpreted with caution. The predictive performance of this model has not been specifically validated in renal SFT populations. Notably, even patients classified as low risk may experience late recurrence or distant metastasis, suggesting that exclusive reliance on this model could underestimate the long term biological aggressiveness of renal SFT. Moreover, the model does not incorporate renal-specific prognostic variables such as surgical margin status or vascular invasion, both well-established determinants of recurrence risk in renal oncology. Therefore we suggest that these variables be prospectively documented in future case series to enable the development of a renal-specific risk stratification model. Accordingly, in clinical practice, the WHO risk stratification should serve as a reference tool rather than the sole basis for prognostic assessment. It should be integrated with tumor anatomical location, completeness of resection, and long term postoperative surveillance to guide individualized evaluation. The prognosis of solitary fibrous tumors (SFTs) is strongly influenced by their anatomical origin and histopathological characteristics (15). Lesions developing in extrathoracic sites, such as the kidney, exhibit a substantially higher propensity for recurrence than those confined to the thoracic cavity. Moreover, SFTs with malignant or borderline histological features are significantly more prone to relapse. Notably, even renal SFTs initially identified as benign may recur after a prolonged disease-free interval, occasionally displaying histological transformation toward a more aggressive form. These observations emphasize the importance of sustained, long-term surveillance in affected patients (15). Complete surgical excision with histologically negative margins (R0 resection) continues to represent the fundamental strategy for optimizing patient outcomes. Evidence from renal SFT cohorts demonstrates that benign lesions seldom recur or metastasize after nephrectomy (52). Nevertheless, despite the generally favorable prognosis of renal SFT, individuals presenting with large tumor size or atypical or malignant histopathological features require extended and meticulous follow-up.

### Clinical insights

The key clinical and pathological characteristics of the 111 reported cases are summarized in **Table 1**, and detailed case-level data are presented in **Table 2**. Based on the aggregated data, several recurring patterns of potential clinical relevance can be identified. These patterns are presented as descriptive observations and do not imply formal statistical inference. Pattern 1: Diagnostic evolution toward STAT6 as the anchor, with utility in atypical variants. A temporal shift in diagnostic practice can be observed from the aggregated data. Cases published before 2015 relied primarily on CD34, CD99, and Bcl-2, often with variable staining patterns. In contrast, from 2015 onward, nuclear STAT6 immunohistochemistry began to be reported in a subset of cases, with its use showing a gradual increasing trend over time, particularly after 2019. As illustrated in **Table 2**, some cases exhibited features that could pose diagnostic challenges: Cases 77, 79, 105, and 106 lacked CD34 expression entirely; Cases 76–79 demonstrated BCOR positivity, which may mimic clear cell sarcoma of the kidney; and Case 107 showed cystic change. In diagnostically complex instances where STAT6 was assessed, its expression was usually positive, reinforcing its utility as a reliable marker when morphology or immunohistochemical profiles deviate from typical findings. This temporal shift also explains the seemingly low overall STAT6 positivity rate of 19.8% in **Table 1**, which largely reflects the absence of STAT6 testing in cases published before 2015 rather than a genuine negative rate. Pattern 2: Favorable outcomes with nephron-sparing surgery in selected patients. The experience summarized in **Table 2** suggests that nephron-sparing surgery may achieve favorable outcomes in appropriately selected patients. Cases 29 (8 cm, upper pole), 62 (2.5 cm), 65 (8 cm, lower pole), 68 (4 cm), and 74 (4 cm, lower pole) all underwent partial nephrectomy with no evidence of disease during follow-up (9 to 36 months). However, given the small sample size and limited follow-up duration, these observations should be interpreted cautiously. Pattern 3: Variable biological behavior across cases. The aggregated data reveal substantial heterogeneity in the clinical course of rSFTs. Case 75 experienced local recurrence within 9 months with subsequent progression. Case 102 progressed to pulmonary and brain metastases within 16 months. Case 64 had local recurrence at 36 months. Cases 49 and 60 developed metastases 96 months after primary surgery, illustrating an indolent course with very late recurrence. Pattern 4: Large tumor size as a potential predictor of adverse outcomes. An association between tumor size and clinical outcome can be derived from the aggregated data. Among cases with documented recurrence, metastasis, or disease-related death, a substantial proportion had tumors exceeding 10 cm in at least one dimension, including Cases 16, 49, 60, 64, 75, and 96. Conversely, patients with tumors <7 cm who underwent complete resection achieved durable disease-free survival with no reported recurrences.

Collectively, these patterns reveal several cross-cutting themes. The diagnostic paradigm has shifted decisively toward confirmation centered on STAT6, a trend especially valuable in cases with atypical morphological or immunohistochemical profiles. Experience with nephron-sparing surgery, although limited, suggests that for exophytic, well-encapsulated tumors in polar locations, partial nephrectomy may offer oncologic control comparable to radical nephrectomy while preserving renal parenchyma. The marked variability in clinical trajectories, ranging from prolonged remission to late recurrence or rapid progression, underscores that risk assessment cannot rely on any single parameter. Tumor size appears to be one such factor; larger tumors show an association with increased risk of adverse outcomes, yet exceptions exist, underscoring that other clinical and pathologic features also contribute to disease progression. Overall, these observations reinforce the necessity of individualized management strategies that integrate diagnostic precision, surgical decision-making, and risk adapted surveillance.

## Limitations and future directions

The current literature on rSFTs carries several inherent limitations that also point toward future research priorities. The evidence base consists predominantly of case reports and retrospective series, with no prospective studies or controlled trials available to support comparative treatment conclusions. The wide variation in reporting across studies also limits meaningful comparison: follow-up duration ranges from 1 month to 9 years, with many cases lacking reported follow-up; immunohistochemical panels differ across studies; and critical variables such as mitotic index, surgical margin status, and tumor necrosis are inconsistently documented. Publication bias is also likely, as unusual presentations or adverse outcomes are preferentially reported. Additionally, the WHO risk stratification model for solitary fibrous tumors, while useful, has not been validated specifically for renal tumors and does not incorporate renal-specific factors such as margin status or tumor anatomical location.

Addressing these limitations will require collaborative efforts with direct clinical implications. An international registry for rSFTs tumors would enable standardized data collection, allowing for more accurate risk stratification and better guidance for postoperative surveillance. Refinement of risk assessment tools specifically for renal tumors, incorporating factors such as tumor size, surgical margin status, and anatomical location, would help clinicians tailor follow-up strategies to individual patients. For aggressive or metastatic cases, molecular profiling may identify potential therapeutic targets, while prospective evaluation of systemic therapies within rare tumor networks is needed to establish treatment options for unresectable or disseminated disease. Finally, consensus-based follow-up guidelines should be developed. The documented cases of late recurrence support surveillance beyond 5 years, with imaging intervals informed by initial tumor size, histologic features, and margin status.

## Conclusion

The rSFT is an uncommon mesenchymal neoplasm exhibiting variable biological behavior. Accurate diagnosis relies on integrated clinical, imaging, histopathological, and immunohistochemical assessment, with nuclear STAT6 expression serving as a key diagnostic marker. Complete surgical resection with negative margins remains the primary treatment approach, whereas nephron-sparing surgery may be considered in carefully selected patients. Although most cases are associated with favorable outcomes, a subset may recur or metastasize, supporting the need for prolonged surveillance. Given that available evidence is largely derived from case reports and retrospective series, findings should be interpreted with caution. Further collaborative studies are warranted to refine risk stratification and guide clinical management.
