# Supplementary material for: Clinical characteristics and management of renal solitary fibrous tumors: a narrative review with literature-derived case evidence
Source: Front Oncol. 2026 Aug 6;16:1894949. doi: 10.3389/fonc.2026.1894949 (PMC13491029; doi:10.3389/fonc.2026.1894949)
Supplement: Supplementary file 1 [file SupplementaryFile1.docx]

**PUBMED:**

(("Solitary Fibrous Tumor"[Mesh] OR "Solitary Fibrous Tumor"[Title/Abstract] OR "Solitary Fibrous Tumors"[Title/Abstract] OR "SFT"[Title/Abstract] OR "SFTs"[Title/Abstract] OR "Hemangiopericytoma"[Mesh] OR "Hemangiopericytoma"[Title/Abstract] OR "Hemangiopericytomas"[Title/Abstract]) AND ("Kidney"[Mesh] OR "Kidney Neoplasms"[Mesh] OR "Renal"[Title/Abstract] OR "Kidney"[Title/Abstract]))

**Web of Science:**

TS=((("Solitary Fibrous Tumor" OR "Solitary Fibrous Tumors" OR "SFT" OR "SFTs" OR "Hemangiopericytoma" OR "Hemangiopericytomas")) AND ("Kidney" OR "Renal" OR "Kidney neoplasms" OR "Renal neoplasms" OR "Kidney tumor" OR "Renal tumor"))

Document type limited to Article or Review Article

Language: English
